# Differences in pre-sleep activity and sleep location are associated with variability in daytime/nighttime sleep electrophysiology in the domestic dog

**DOI:** 10.1038/s41598-018-25546-x

**Published:** 2018-05-08

**Authors:** Nóra Bunford, Vivien Reicher, Anna Kis, Ákos Pogány, Ferenc Gombos, Róbert Bódizs, Márta Gácsi

**Affiliations:** 1 Eötvös Loránd University, Institute of Biology, Department of Ethology, 1117 Budapest, Hungary; 2 Hungarian Academy of Sciences, Institute of Cognitive Neuroscience and Psychology, 1117 Budapest, Hungary; 3 Budapest University of Technology and Economics, Department of Cognitive Science, 1111 Budapest, Hungary; 4 Pázmány Péter Catholic University, Faculty of Humanities and Social Sciences, 2087 Piliscsaba, Hungary; 5 Semmelweis University, Institute of Behavioural Sciences, 1089 Budapest, Hungary; 6 MTA-ELTE Comparative Ethology Research Group, 1117 Budapest, Hungary

## Abstract

The domestic dog (*Canis familiaris*) is a promising animal model. Yet, the canine neuroscience literature is predominantly comprised of studies wherein (semi-)invasive methods and intensive training are used to study awake dog behavior. Given prior findings with humans and/or dogs, our goal was to assess, in 16 family dogs (1.5–7 years old; 10 males; 10 different breeds) the effects of pre-sleep activity and timing and location of sleep on sleep electrophysiology. All three factors had a main and/or interactive effect on sleep macrostructure. Following an active day, dogs slept more, were more likely to have an earlier drowsiness and NREM, and spent less time in drowsiness and more time in NREM and REM. Activity also had location- and time of day-specific effects. Time of day had main effects; at nighttime, dogs slept more and spent less time in drowsiness and awake after first drowsiness, and more time in NREM and in REM. Location had a main effect; when not at home, REM sleep following a first NREM was less likely. Findings are consistent with and extend prior human and dog data and have implications for the dog as an animal model and for informing future comparative research on sleep.

## Introduction

The domestic dog (*Canis familiaris*) is a potentially reliable and valid animal model of complex – such as cognitive and social – human functions^1–3^, as domestication allowed for dogs to adapt to an environment shared with humans^4^. Dogs have been studied in comparative research on behavior, cognition, and emotion, such as in studies of attachment^5,6^, inequity aversion^7^, automatic^8^ and selective imitation^9^, sensitivity to ostensive cues^10,11^, and social referencing^12^. More recently, behavioral approaches are being combined with neuroscience methods to study awake dog behavior including reward-^13^ and visual^14,15^ processing, memory consolidation^16^ and stimulus discrimination^17,18^.

Two limitations of the available canine neuroscience literature are that it is predominantly comprised of studies with (semi-)invasive^17,18^ or intensive training methods (e.g., evoked response potentials [ERP]^14,15^; functional magnetic resonance imaging [fMRI]^19,20^) and focused on behavior of awake dogs. As such, little is known about the electrophysiology of sleep in dogs (or, for that matter, about sleep in any species other than humans and rodents, and thus about relevant evolutionary questions). Except for a few recent studies^16,21–23^, most relevant data has been obtained during chemically-induced sleep and/or with intracranial electrodes^24–26^. Because of differences between chemically-induced and natural sleep^27^ and that intracranial electrodes can be used only with a restricted subgroup (e.g., those requiring a neurological evaluation/intervention or laboratory-bred and -kept animals), these findings are with limited generalizability. Beyond sleep in dogs, even less is known about the different factors that may influence it. There appears utility in extending evaluations of the dog as an animal model from awake to sleep behavior as existing data suggests noteworthy comparability across dog and human sleep. Dogs’ circadian rhythm is similar to humans’ and although their sleep is polyphasic (i.e., during the day, active behavior and sleep alternate), they have been shown, similar to humans, to exhibit greatest sleep propensity and spend more time in non-rapid eye movement (NREM) and rapid eye movement (REM) sleep during the night^28,29^ in invasive laboratory studies.

Sleep is not only a scientifically but a practically important area of inquiry as it serves and supports important physiological, cognitive, and behavioral functions^30^. Differences in sleep are associated with differences in behavior (e.g., aggression, hyperactivity, impulse dyscontrol)^31,32^, cognition (e.g., attention, learning, memory consolidation)^33^, emotion regulation^31,32^, physical health (e.g., cardiovascular, endocrine, metabolic, and immune function), and quality of life^34^.

To begin addressing the noted limitations to the literature, our group developed a non-invasive canine polysomnography (PSG) method that allows for simultaneous recording of physiological variables including neural oscillations produced by electroencephalography (EEG)^21^. This method is ideal to complement behavioral with neural data on untrained pet dogs and thus to address a shortcoming of the available dog and human sleep literature, which is variability in research design and methodology, without sufficient consideration of the different factors that may influence sleep. These factors are timing of sleep (i.e., during the day or during the night), extent of pre-sleep activity, and location of sleep (the familiarity of the sleeping environment).

Timing of sleep influences qualitative and quantitative indices of sleep; in humans, nighttime relative to daytime sleep is associated with shorter sleep latency and more time in deep sleep^35^. Beyond nighttime relative to daytime differences, sleep structure also differs between the first and second halves of the night, e.g., stages 3 and 4 (i.e., deep sleep characterized by high voltage, slow EEG activity with some spindling superimposed) have been shown to decrease markedly and REM to increase considerably from the first to the second half of the night^36,37^. Of note, these tendencies are not present in daytime sleep or are – as is the case with REM sleep – present but in the opposite direction (i.e., REM decreases over time)^38^. In addition, other data show that timing of sleep also affects various cognitive processes (e.g., memory consolidation)^39^. Relevant findings have also been obtained with rodents, where the same amount of sleep deprivation (6 hrs) differentially impacts learning (i.e., fear conditioning) depending on its timing, i.e., whether it occurs in the sleep vs. the waking period^40^. Yet, all *non*-invasive canine PSG data have been obtained during the afternoon^16,21^, indicating need for research comparing the effects of timing on quantitative indices of sleep.

With regard to pre-sleep activity, data examined across three meta-analyses^41–43^ indicate that acute exercise has a small positive effect on total sleep time, sleep onset latency, sleep efficiency, stage 1 sleep, and slow wave sleep; a moderate positive effect on wake time after sleep onset; and a small positive effect on REM sleep. Regular exercise has a small positive effect on total sleep time and sleep efficiency; a small-to-medium positive effect on sleep onset latency; and a moderate positive effect on sleep quality. In humans, these effects are moderated by age, sex, baseline physical activity level, as well as exercise duration, time, type, and adherence^43^. In dogs, an active relative to a passive day has been shown to be associated with changes in sleep macrostructure such as decreased sleep latency, increased time spent asleep, and increased time spent in NREM^21^.

Finally, related to the location of sleep, qualitative indices suggest that at home, relative to a novel environment, better quality and greater quantity of sleep is obtained and this sleep reduces fatigue more effectively^44^. Quantitative indices of sleep suggest sleeping in a familiar environment is associated with shorter sleep latency and longer sleep duration as well as shorter time spent in drowsiness and longer time spent in REM compared to sleeping in the laboratory^45^. Further, a first-night effect (a universally observed ‘artefact’ resulting from the difference between the first and second sleep recording)^46,47^ is less common in home studies than in laboratory studies^48^. Yet, all available canine PSG data have been obtained in the laboratory^16,21^.

Taken together, prior findings with humans indicating an effect of timing and location and with dogs indicating an effect of activity, a key next step is examining, both during daytime and during nighttime sleep, whether factors such as activity and location of sleep, on their own and/or in combination with timing and each other (i.e., the factors interact), influence quantitative indices of dogs’ sleep. Accordingly, our goal in the current study was to examine the effects of (1) timing (2) activity before sleep and (3) location of sleep and/or (4) interactions among these on sleep physiology in the dog, indexed by sleep macrostructure.

Of note, choice of quantitative indices of sleep was informed by three primary considerations. First, the chosen macrostructural variables (see *Method, Data Analysis*) are the most commonly used and well-validated quantitative indices of sleep health and quality^49^. Second, prior human and dog literature indicate that timing, activity, and location of sleep has an effect on these macrostructural variables^21,41–43^. Equally informatively, the chosen variables are clinically and practically meaningful given their sensitivity to differences in age^50^ and to differences in disease status including epilepsy^51,52^, attention-deficit/hyperactivity disorder^53^, autism spectrum disorder and related disorders^54,55^, and depression^56,57^ as well as association with subjective indices of sleep^58^.

## Results

### Sleep macrostructure

#### Proportion of time spent sleeping

Time of day and activity both influenced proportion of time spent sleeping, separately (GLMM; χ^2^_daytime_(1) = 4.165, *p* = 0.041, and χ^2^_activity_(1) = 10.878, *p* < 0.001), because dogs slept more at nighttime (day → night: *b* = 0.296 [0.012; 0.580] and after an active day (typical → active day: *b* = 0.690 [0.326; 1.056]; see online supplement, Table S1, Fig. S1).

#### Proportion of time spent awake after first drowsiness

Proportion of time spent awake after first drowsiness was influenced by time of day (GLMM; χ^2^(1) = 6.474, *p* = 0.011), period (χ^2^(5) = 11.337, *p* = 0.045), and technical difficulties (χ^2^(2) = 7.927, *p* = 0.019). In addition, activity had location-specific effects (χ^2^_activity x location_(1) = 3.967, *p* = 0.046) (see online supplement, Fig. S2a–c). Dogs spent less time awake after first drowsiness at nighttime (day → night: *b* = −0.440 [−0.777; −0.103]). For differences in proportion of time awake depending on period, see online supplement, Table S2. Dogs spent more time awake after minor (no → minor: *b* = 0.482 [−0.270; 1.234]) or major (no → major: *b* = 1.686 [0.460; 2.912]) technical difficulties. When measured at home, there was no difference given activity level on proportion of time awake after first drowsiness but when measured not at home, dogs spent less time awake following an active day (not home vs. home, typical → active day: *b* = −0.578 [−1.139; −0.018]).

#### Proportion of time spent awake after first non-drowsiness sleep

Activity had location-specific effects (GLMM; χ^2^_activity x location_(1) = 5.283, *p* = 0.022) on proportion of time spent awake after first non-drowsiness sleep (see online supplement, Table S3, Fig. S3). When measured at home, there was no difference in proportion of time awake after first non-drowsiness sleep depending on activity level but when measured not at home, dogs spent less time awake after first non-drowsiness sleep following an active day (not home vs. home, typical → active day: *b* = −0.860 [−1.578; −0.143]).

#### Latency to first drowsiness

Latency to first drowsiness sleep was affected by activity (MECM; χ^2^(1) = 5.024, *p* = 0.025) in that dogs were more likely to have an earlier drowsiness following an active day (typical → active day: exp(*β*) = 4.877 [1.225; 19.413], see online supplement, Table S4, Fig. S4).

#### Latency to first non-drowsiness sleep

Latency to first non-drowsiness sleep was affected by activity (MECM, χ^2^(1) = 6.375, *p* = 0.012) in that dogs were more likely to have an earlier non-drowsiness sleep following an active day (typical → active day: exp(*β*) = 3.047 [1.315; 7.062]), see online supplement, Table S5, Fig. S5).

#### Proportion of time in drowsiness

**Figure 1:**
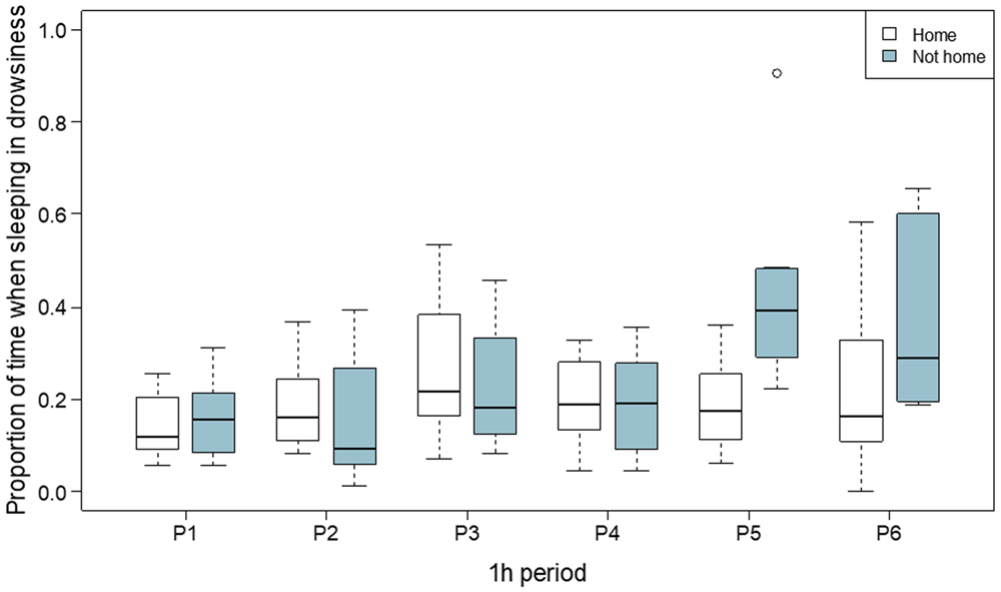
Location-specific effects of period on proportion of time spent in drowsiness: Period had location-specific effects on proportion of time spent in drowsiness, such that the difference between time in drowsiness during the fifth period was greatest (and positive) when measured not at home. Note. The figure illustrates data from nighttime recordings only.

Proportion of time spent in drowsiness was influenced by activity (GLMM; χ^2^(1) = 7.532, *p* = 0.006) and time of day (χ^2^(1) = 20.917, *p* < 0.001, see online supplement, Fig. S8). In addition, period had location-specific effects (χ^2^_location x period_(5) = 21.649, *p* < 0.001). Dogs spent less time in drowsiness after an active day (typical → active day: *b* = −0.085 [−0.145; −0.026]) and at nighttime relative to daytime (day → night: *b* = −0.098 [−0.143; −0.053]). With regard to the interaction between period and location, there were differences between periods and differences in these differences given measurement location. For example, the difference in time in drowsiness was greatest between the third and fifth periods and when measured not at home (not home vs. home, period 1 → period 5: *b* = 0.240 [0.106; 0.374], Fig. 1). For additional detail on differences in proportion of time in drowsiness given location-specific effects of period and the effects of activity and time of day on proportion of time in drowsiness, see online supplement, Table S6 and Fig S6, respectively).

#### Proportion of sleeping time in NREM

**Figure 2:**
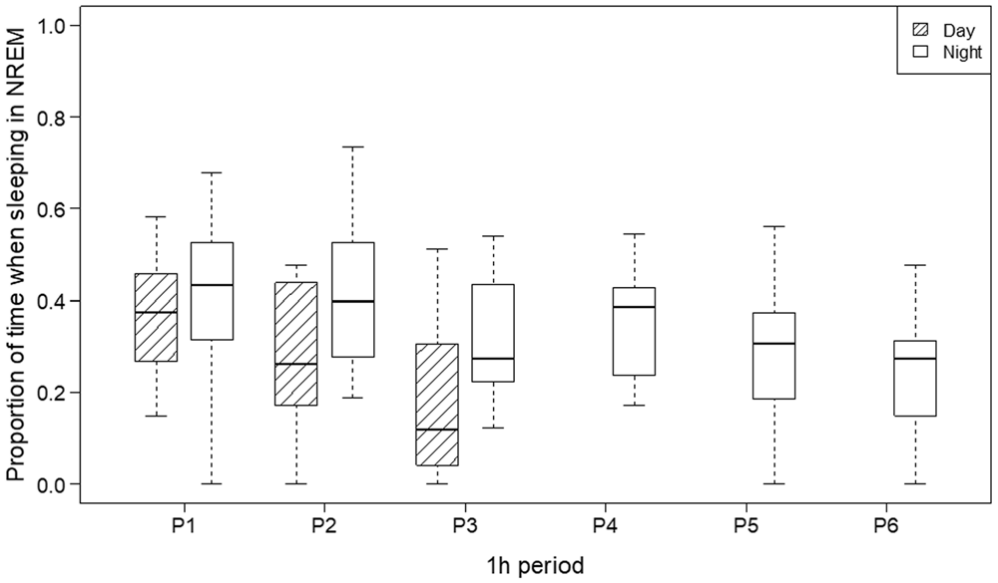
The effects of period and daytime on proportion of sleeping time in NREM: Dogs spent more time in NREM at nighttime and time in NREM decreases over time, both during daytime and during nighttime sleep.

Proportion of time spent in NREM was influenced by activity (GLMM; χ^2^(1) = 11.714, *p* < 0.001), period (χ^2^(5) = 36.236, *p* < 0.001), time of day (χ^2^(1) = 18.390, *p* < 0.001), and technical difficulties (χ^2^(2) = 8.234, *p* = 0.016, see online supplement, Figs S9 and S10). Dogs spent more time in NREM after an active day (typical → active day: *b* = 0.123 [0.061; 0.184]) and at nighttime (day → night: *b* = 0.111 [0.062; 0.161]). With regard to the effect of period, the greatest difference from time in NREM during the first period was with time in NREM during the third (period 1 → period 3: *b* = −0.161 [−0.226; −0.096]) and sixth periods (period 1 → period 6: *b* = −0.214 [−0.299; −0.129], Fig. 2). Moreover, dogs spent less time in NREM after minor (no → minor: *b* = −0.069 [−0.183; 0.044]) or major (no → major: *b* = −0.262 [−0.445; −0.077]) technical difficulties. For additional detail on differences in proportion of time in NREM period and on effects of activity, technical difficulties, on proportion of observation time spent in NREM, see online supplement, Table S7, Fig. S7a,b).

#### Proportion of sleeping time in REM

**Figure 3:**
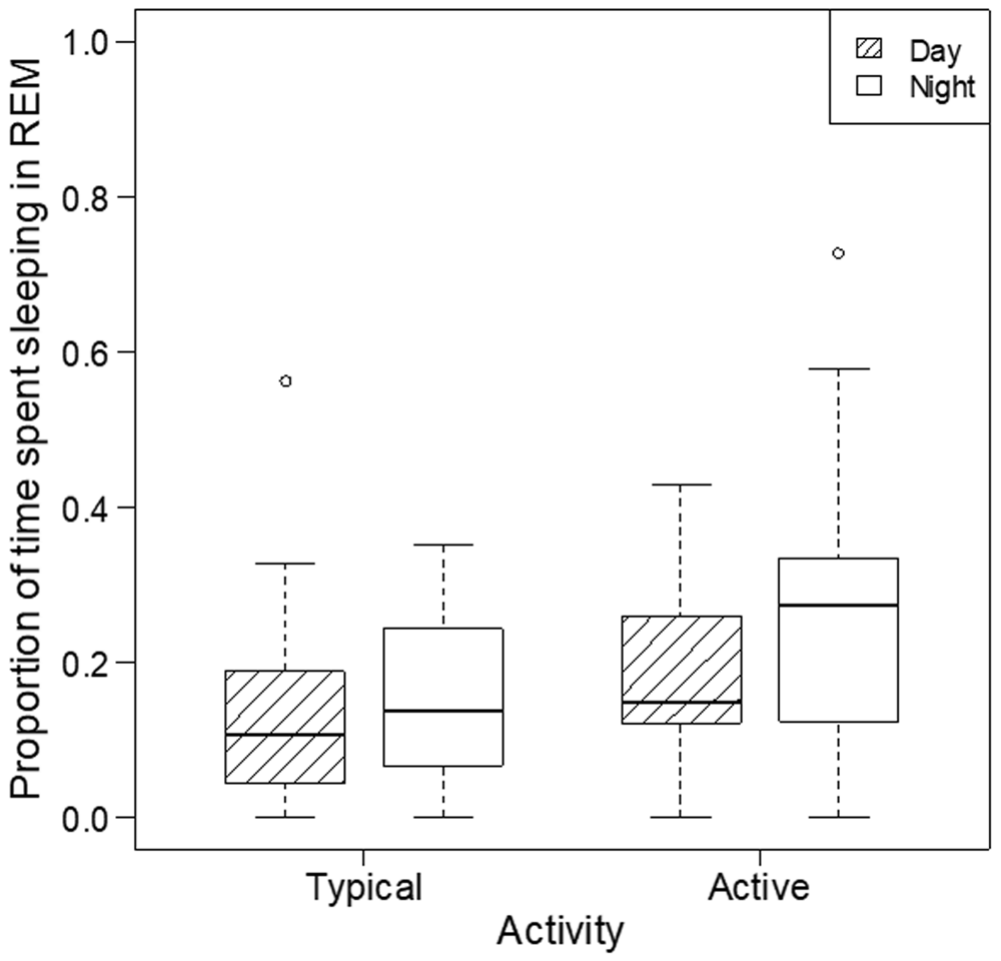
The effects of daytime and activity on proportion of sleeping time in REM: Dogs spent more time in REM after an active day and at nighttime.

Proportion of time spent in REM was influenced by activity (ORM; χ^2^(1) = 4.641, *p* = 0.031) and time of day (χ^2^(1) = 5.415, *p* = 0.020). Dogs spent more time in REM after an active day (typical → active day: *b* = 2.989 [1.169; 7.639]) and at nighttime (day → night: *b* = 2.158 [1.123; 4.146], Table S8, Fig. 3).

#### Latency to first REM after first drowsiness

Activity had time of day-specific effects (MECM; χ^2^_activity x daytime_(1) = 6.292, *p* = 0.012) on latency to first REM (see online supplement, Table S9, Figure S9). The interaction was driven by an earlier REM sleep after drowsiness during nighttime as opposed to daytime sleep after an active day (night vs. day, typical → active day: exp(*β*) = 9.786 [1.538; 62.250]).

#### Latency to first REM a°ter first non-drowsiness sleep

Latency to first REM after first non-drowsiness sleep was influenced by location (MECM; χ^2^(1) = 8.700, *p* = 0.003, see online supplement, Table S10, Fig. S10a,b) and activity had time of day-specific effects (χ^2^_activity x daytime_(1) = 4.176, *p* = 0.041, see online supplement, Fig. S13). When not home, REM sleep after first non-drowsiness was less likely (home → not home: exp(*β*) = 0.140 (0.038; 0.512), but it was more likely during nighttime as opposed to daytime after an active day (night vs. day, typical → active day: exp(*β*) = 5.658 [1.032; 30.992]).

## Discussion

Prior human data indicate differences in quantitative indices of sleep depending on timing^36,37^ and location^45,48^. Existing human and canine data indicate that sleep depends on pre-sleep activity^16,21,41–43^. Yet, neither the unique nor the interactive effects of timing or location – nor the interactive effects of pre-sleep activity with timing and location – on sleep had been examined in dogs.

Our findings indicate complex relations among influencing factors. With regard to analyses of sleep macrostructure, findings indicated that dogs slept more and spent more time in NREM at nighttime and after an active day, consistent with previously published human and dog data^16,21,41–43^. Dogs also spent more time in REM at nighttime and after an active day. The latter result is dissimilar from earlier findings indicating no differences in REM duration following an active relative to a passive day^21^. Differences in the way in which “active” was defined across the two studies (i.e., any competition or advanced training in the current study vs. 6–8 hours total of sleepless activity mainly involving 4–6 hours of excursions/walking in^21^) and corresponding potential differences in emotional load might account for this discrepancy as REM sleep is involved in processing emotional memories^59^. Time spent in NREM and REM sleep are key quantitative indices of sleep as altered sleep architecture has been shown to be associated with neurodevelopmental and psychiatric disorders^51–53^. As such, our results indicating that the time dogs spent in NREM and REM was sensitive to timing and activity have potential importance and usefulness in areas of research focused on those disorders.

After an active day there was a greater difference between nighttime and daytime latency to first REM both after first drowsiness and after first non-drowsiness sleep, in that there was earlier emergence of REM sleep after drowsiness during nighttime as opposed to daytime sleep after an active day. When not at home, the probability of having reached the REM phase following a given time after first non-drowsiness was lower. Similar to time spent in NREM and REM sleep, latency to REM sleep is not only sensitive to effects of timing, activity, and location but is sensitive to differences in subjective indices of sleep^58^, considered a biological marker of – or implicated in – several psychiatric disorders^49,53,55^.

Dogs spent more time awake after their first drowsiness sleep at nighttime, across all periods. When measured at home, there was no difference given activity level on proportion of time awake after first drowsiness, but when measured in a location other than their homes, dogs spent less time awake following an active day. Dogs also spent less time awake after their first non-drowsiness sleep following an active day, but as with time awake after first drowsiness sleep, only when measured in a location other than their homes. Together, these data indicate that the effects of activity are apparent on time spent awake after first drowsiness and first NREM only when measured in an unfamiliar location. Increased time spent awake after first drowsiness and first non-drowsiness is negatively associated with age. Our results indicate that, in research with the dog as an animal model of or age-related differences, sleep measurements may be most informative if conducted in a location other than dogs’ homes.

Also consistent with prior human data, the probability for dogs to reach first drowsiness and a first non-drowsiness sleep after a given time elapsed was higher following an active day^43^. In line with conceptualization of time spent in drowsiness characterized by alpha activity that indexes superficiality of sleep^60^, dogs spent less time in drowsiness at nighttime and after an active day. Earlier findings on dogs indicate no difference in drowsiness duration following an active relative to a passive day^21^, likely due to the typical day used in the present study vs. the passive day used in the prior study (e.g., when subjects had a passive 6–8-hour pre-sleep period they might have spent most of this time in drowsiness, increasing their propensity for drowsiness compared to an active day).

Interestingly, although the impact of location of sleep in dogs has not been examined prior to the current study, earlier data obtained in a relevant research suggested that dogs sleeping indoors spent 80%, dogs sleeping outdoors in a yard spent 70%, and dogs sleeping outdoors in a non-fenced area spent 60% of the night asleep^61^. While familiarity with location of sleep was not manipulated in this prior study, differences between the indoor vs. outdoor environment may index the number of novel stimuli with which dogs are confronted as well as protectedness during sleep, the impact of which may be subserved by mechanisms similar to those subserving the present findings on the impact of location on dogs’ sleep.

As noted, findings indicate complex relations both among influencing factors of interest and among these and technical difficulties. As these results were obtained with a relatively small sample of 16 dogs, they indicate that the factors we considered and their inter-relationship are robustly related to sleep, suggesting it will be important to account or systematically control for all in future studies. Other environmental factors, such as the absence/presence of other humans and/or dogs may have an effect on dogs’ sleep.

The data obtained here has implications for further validating the dog as an animal model of sleep and thus may inform comparative and translational research on sleep disorders and the prevention and treatment thereof. With regard to implications, it is important to first note that effective use of animals to study typical and disordered sleep must consider differences and similarities between human and animal sleep, as the cyclical organization, daily duration, diurnal timing, and other, e.g., electrophysiological features of sleep extensively vary across^62–64^ and within^65,66^ species. For example, some species exhibit unihemispheric sleep and others spend relatively less^67^ or, as some findings indicate, in case of certain monotremes, no time in REM^62,68,69^ (but see^70^ for a report of adult echidnas exhibiting REM at temperatures within their thermoneutral zone**)**. Relative to birds and mammals, amphibians and reptiles exhibit cortical activity with larger amplitudes during wakefulness than during inactivity and no features characteristic of REM^62,71^. These differences restrict the number of species that are appropriate for modelling human sleep.

Comparing general characteristics of sleep in humans to such characteristics in the most commonly used model species (with the exception of nonhuman primates, in some but not all regards^72^), it is evident that human sleep is better approximated by dog sleep. Specifically, humans differ from rats, mice, cats, and dogs in that human sleep pattern is monophasic/diphasic whereas rat, mouse, cat, and dog sleep is polyphasic and that length of a human sleep bout is 6–8hs, compared to much shorter sleep bouts in rats and mice (less than 15 mins) and cats and dogs (78 mins and 45 mins, respectively)^72^. Conversely, humans are more similar to dogs and cats in that humans’, cats’, and dogs’ primary diurnal sleep phase is in the dark (mice’s and rats’ is in the light) and humans are more similar to dogs with regard to daily sleep duration (daily sleep duration in humans is ~8hs, in dogs it is 8–14hs whereas in mice, rats, and cats, it is longer, typically 12–15hs)^72^. Together, these data indicate that of the most commonly used model species, human sleep is most comparable to dog sleep given the above indices. These similarities, in combination with dogs’ natural cooperativeness and trainability that allow for use of the non-invasive PSG method, suggest the domestic dog is a promising model of human sleep. Although we did not compare across species, our current results further validate the family dog as one such model, insofar as they are consistent with both prior human^41–43,73^ and dog^16,21,29,74^ data (though note two exceptions where we found differences given pre-sleep activity that were not observed in an earlier study^21^). In addition to replication of earlier results on effects of pre-sleep activity on canine brain electrophysiology, this is the first characterization of the effects of location and timing on dogs’ sleep, thereby contributing to the rapidly growing area of canine (neuro)cognitive science. The next generation of studies may aim to determine whether quantitative indices of sleep are modulated by individual variability similar to humans where, e.g., the effect of activity on sleep is moderated by age, sex, baseline physical activity level, as well as exercise duration, time, type, and adherence^43^.

## Methods

### Subjects

Subjects were *N* = 16 family dogs (1.5–7 years old; 10 males; from 10 different breeds and 2 mongrels). Owners were recruited either from the Family Dog Project (Eötvös Loránd University, Department of Ethology) database or because they were acquainted with research staff. All experimental protocols were approved by the Állatkísérleti Tudományos Etikai Tanács (Scientific Ethics Committee for Animal Experimentation) of Budapest, Hungary and carried out in accordance with the relevant guidelines and regulations.

Participating in the current research did not require prior training. All subjects were measured on three occasions. First, a 3-hour-long afternoon recording was conducted in the sleep laboratory to adapt dogs to measurement conditions^46^ (adaptation data is not analyzed herein). The second and third occasions were a 3-hour-long afternoon nap and a 6-hour-long night recording in a counterbalanced order. For a given subject both the afternoon and the nocturnal sleeps were measured after a similarly active day (either an active or a typical one) and at the same location (either at home or at a different place, which was not the laboratory where the adaptation measurement was done). Each dog thus had an adaptation, a daytime, and a nighttime measurement. Four dogs were measured after an active day (i.e., a physically and mentally loaded day such as due to competition or advanced training), at home; four after a typical day (i.e., the dog did not have a highly active or loaded day), at home; four after an active day, not at home (e.g., a camp, a friend’s place, or the sleep laboratory); and four after a typical day, not at home.

### Procedure

**Figure 4:**
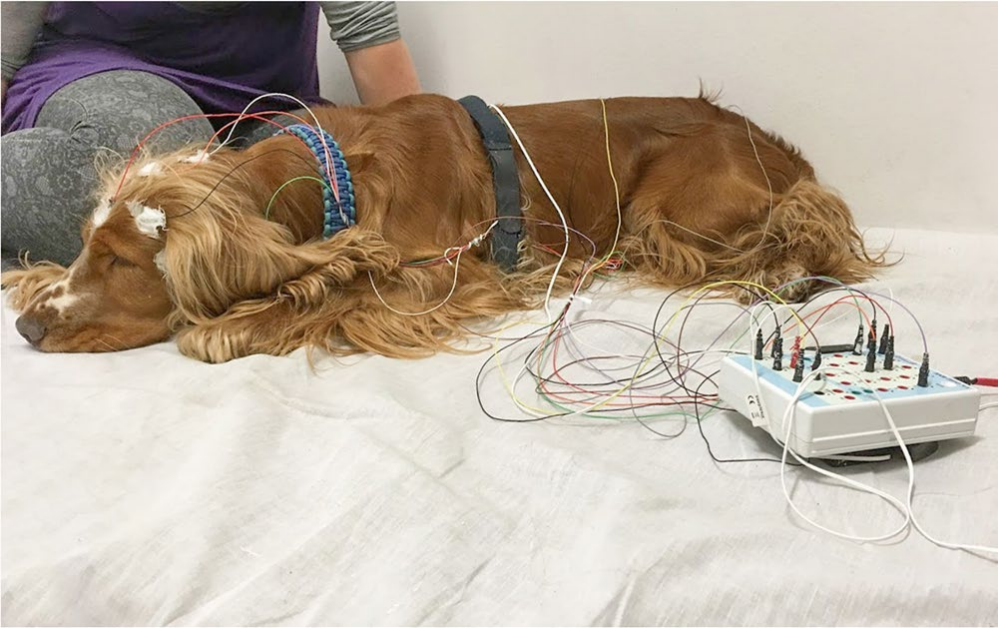
Photo of a dog with electrode placement. Electrode placement involved attachment of EEG and EOG electrodes on the scalp, ECG electrodes bilaterally over the second rib, and EMG electrodes bilaterally on the musculus iliocostalis dorsi. Respiration was recorded via a chest respiratory belt.

Sleep was monitored by PSG, simultaneously recording neural oscillations (EEG), electrooculogram (EOG), electrocardiogram (ECG), respiration, and electromyography (EMG). Following prior research^21^, electrode placement involved attachment of scalp electrodes over the anteroposterior midline of the skull (Fz, Cz, Pz) and on the left zygomatic arch (*os* zygomaticum; F7). The Fz-Cz derivation served as the EEG signal, the F7-Cz derivation served as the EOG signal. The ground electrode (G) was placed on the left musculus temporalis. Gold-coated Ag|AgCl cup electrodes fixed with EC2 Grass Electrode Cream (Grass Technologies, USA) were used. All scalp electrodes were placed on a bone to minimize muscle tone and movement artifacts. ECG electrodes were placed bilaterally over the second rib and EMG electrodes were placed bilaterally on the musculus iliocostalis dorsi. Respiration was recorded via a chest respiratory belt. See Fig. 4 for photo of a dog with electrode placement. Signals were collected, prefiltered, amplified and digitized at a sampling rate of 1024 Hz/channel by using the SAM 25 R style MicroMed Headbox (MicroMed Inc, Houston, TX, USA), with hardware passband at 0.5–256 Hz, sampling rate of 512 HZ, anti-aliasing filter with cut-off frequency at 1 kHz, and 12-bit resolution covering a voltage range of±2 mV as well as second-order software filters at 0.016 Hz (high pass) and 70 Hz (low pass) using System Plus Evolution software (MicroMed Inc, Houston, TX, USA). Impedances for the EEG electrodes were kept below 20 kΩ.

Dogs were allowed to explore the room for five to ten minutes, after which their owners assisted research staff with placing surface electrodes on the dog. During electrode placement, all dogs were reinforced using social reinforcement (e.g., petting, praise) and some were also reinforced using treats. Owners were asked to turn off radio-frequency signal transmission on their cellphones, take place on the mattress (with their dog either on the mattress or on the floor next to it), and engage in a quiet activity, e.g., read, sleep, or watch a movie on a laptop (again, with radio-frequency signal transmission suspended) with earphones during measurements. Once the dog and owner were comfortable, research staff exited the room and monitored the measurement on a laptop. In case of home measurements, the owner’s partner or roommate as well as other dogs may have also been present in the room; this was the case in 9 out of 16 dogs, mostly during nighttime measurements. These individuals and other dogs were quiet during measurements. In case of displacement or malfunction of electrodes (based on visual inspection of the live EEG signal), research staff re-entered the room and either re-placed or changed the electrode (this occurred on <10% of the occasions across all 48 measurements).

To test the effects of the factors of interest (timing of sleep, pre-sleep activity, location of sleep) PSG recordings were carried out in the following conditions.

### Timing of sleep

Following adaptation, dogs participated in a three-hour-long daytime sleep measurement (starting between noon and 6:00 pm, following Kis *et al*., 2014) and a six-hour-long nighttime sleep measurement (in line with the owner’s and dog’s usual bedtime, starting between 10 pm and 1am). Timing of sleep was a within-subjects factor.

### Activity

For our purposes, an active day meant that the dog had a physically and mentally loaded day (e.g., competition, advanced training at dog school) and a typical day meant that the dog did not have a loaded or highly active day. Activity was a between-subjects factor; dogs had either an active or a typical day both before the daytime and nighttime recording.

### Location of sleep

Home measurements were conducted at the owners’ homes, where the dogs typically slept. The not-at-home sleep took place at various unfamiliar locations (e.g., a camp, a friend’s place, or the sleep laboratory) that were different from both the home and the adaptation locations. The University sleep laboratory was set up as an ordinary room, similar to the other non-home locations, with a mattress on the floor and a blanket. Location of sleep was a between-subjects factor; dogs slept either at home or not at home both during the daytime and the nighttime recordings.

In addition to the named manipulated factors of interest, degree of technical difficulties experienced was recorded and examined as an additional, non-manipulated factor given reason to believe that these may have also had an effect on variability in sleep macrostructure. Technical difficulties were defined as having any sort of problem in-between completion of electrode placement and the start of recording (e.g., need for re-placement of electrodes, weak signal from electrodes, recording did not start). No difficulties meant that the time in-between completion of electrode placement and start of recording was ≤10 mins (*n* = 24). Minor difficulties meant that the time in-between completion of electrode placement and start of recording was <10 mins ≤30 mins (*n* = 6). Major difficulties meant that the time in-between completion of electrode placement and start of recording was <30 mins ≤60 mins (*n* = 2).

### Data analysis

Sleep recordings were visually scored by an experienced sleep researcher (VR) in accordance with standard criteria^29,75^ adapted for dogs (Kis *et al*., 2014). For inter-rater reliability, a second experienced sleep researcher (AK) also scored 5 dog measurements (39 epochs/recording). Reliability was almost perfect (Cohen’s κ = 0.93). Of all recordings of all dogs, only one, 12-minute portion of one recording was of insufficient quality for analysis, due to ECG signal superimposing on the EEG signal (the rest of the 6-hour recording was clean and rich in sleep stages).

**Figure 5:**
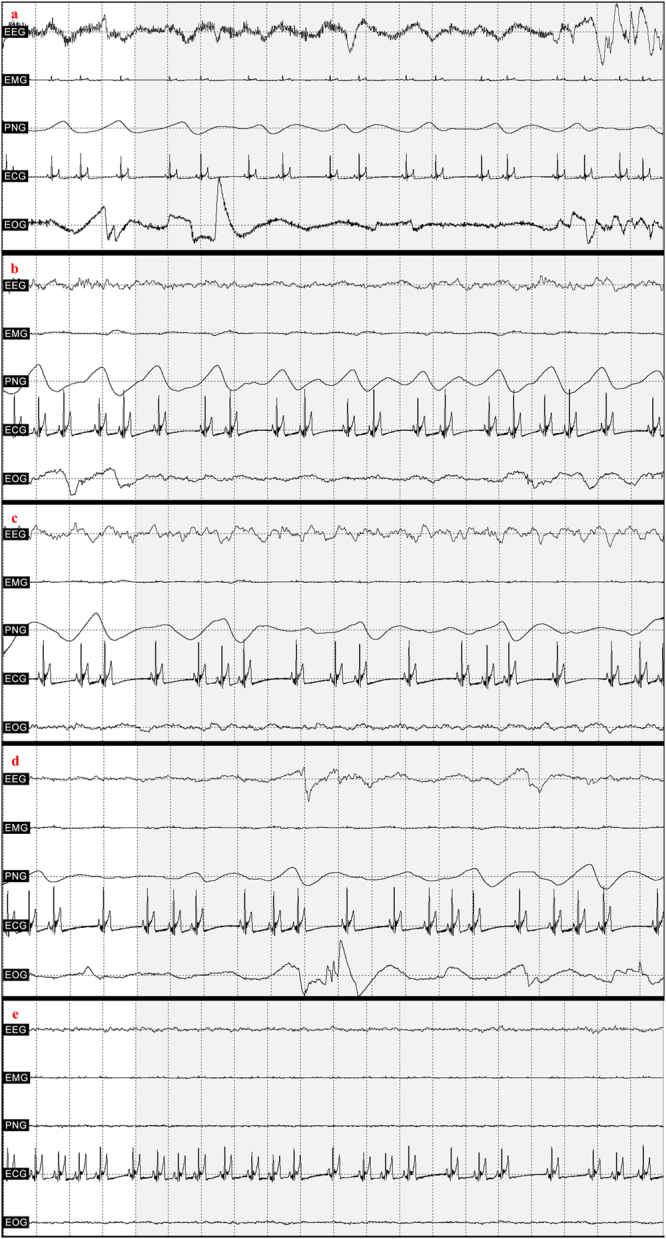
Representative EEG traces from the different sleep stages*. Note*. a = wake, b = drowsiness, c = NREM, d = REM with eye movement, e = REM without eye movement.

A program developed by our laboratory (Fercio’s EEG Plus, © Ferenc Gombos 2009–2016) was used to analyze and export data. The program divides each measurement into 20-sec segments, which were manually scored and provided data for exporting macrostructural variables (see Fig. 5 for a visual depiction of representative EEG traces from the different sleep stages).

The following variables were examined as independent variables. Time of day (factor with two levels: day or night), period (factor of 1-hour periods; 1–3 during days and 1–6 during nights), location (factor with two levels: home or not home), activity (factor with two levels: typical or active), technical difficulties (factor with three levels: none or minor or major). For outcomes involving proportions, proportion of observation time and proportion of sleeping time were the numerators to obtain the following dependent variables: proportion of observation time spent sleeping (hereafter: proportion of time spent sleeping), proportion of observation time spent awake (hereafter: proportion of time spent awake) after first drowsiness and after first non-drowsiness sleep, proportion of time spent in drowsiness, NREM, and REM phases. Additional dependent variables of interest were latency to first drowsiness, first NREM, REM after first drowsiness and REM after first non-drowsiness sleep.

### Analytic Plan

Assumptions of statistical tests were considered prior to the analyses. Proportions were logit-transformed, whereas power spectra variables were log-transformed to normalize residual distributions, with the exception of proportion of sleeping time in REM (see below).

Statistical analyses were conducted in R 3.2.3^76^. Models involving proportion dependent variables were analyzed in General Linear Mixed Models (GLMMs) and latency dependent variables were analyzed using Mixed Effects Cox Models (MECMs; R package ‘coxme’,^77^ with occurrence of a sleeping as terminal event. Because distribution of residuals could not be normalized, proportion of sleeping time in REM phase was divided into quartiles and analyzed in Ordinal Regression Models (ORMs; R package “ordinal”)^78^.

In all above models estimating the effects of predictors on indices of sleep macrostructure, full models included the named independent variables with two levels (but technical difficulties with three levels and period with 6 levels) and all two-way interactions. Backwards model selection was based on Akaike information criterion (AIC) values (a model was considered better in case of delta AIC ≥ 2) and the effects of explanatory variables were analyzed by likelihood ratio tests: we provide χ^2^ and *p* values of likelihood ratio tests of models with and without the explanatory variable. For GLMMs and ORMs, parameter estimates (B) whereas for MECMs, hazard ratios (exp[*β*]) with 95% confidence intervals are provided between levels of a given significant fixed effect.

### Exploratory Analyses

Data on sleep EEG power spectrum were derived and signal power spectrum was analyzed as a dependent variable of interest in models similar to those involving macrostructural variables. Information on corresponding methods and results are appended as Supplementary Information (see online supplement, Exploratory Analyses on Sleep EEG Spectrum).

### Data Availability

The datasets generated and/or analyzed in the current study are available from the corresponding author on reasonable request.

## Electronic supplementary material

Supplementary Information
