# Supplementary material for: Differences in pre-sleep activity and sleep location are associated with variability in daytime/nighttime sleep electrophysiology in the domestic dog
Source: Sci Rep. 2018 May 8;8:7109. doi: 10.1038/s41598-018-25546-x (PMC5940857; doi:10.1038/s41598-018-25546-x)
Supplement: Supplementary file 1 — Supplementary Information [file 41598_2018_25546_MOESM1_ESM.docx]

SUPPLEMENTARY INFORMATION to:

Differences in pre-sleep activity and sleep location are associated with variability in daytime/nighttime sleep electrophysiology in the domestic dog

Nóra Bunford, Vivien Reicher, Anna Kis, Ákos Pogány,

Ferenc Gombos, Róbert Bódizs and Márta Gácsi

*Statistical models and abbreviations*

General Linear Mixed Model – GLMM
Mixed Effects Cox Model – MECM
Ordinal Regression Model – ORM

*Explanatory variables and factor levels in models of sleep macrostructure*

Time of day (DT): factor with two levels (day – 1; night – 2)

Period (P): factor with 6 levels (1-3 during days, and 1-6 during nights)

Pre-sleep activity (A): factor with two levels (typical – 1; active – 2)

Locations (Loc): factor with two levels (home – 1; not home – 2)

Activity (A): factor with two levels (typical – 1; active – 2)

Technical difficulties (TD): factor with three levels (no – 0; minor – 1; major – 2)

*Table S1*

Variables included in the final GLMM of proportion of observation time spent sleeping. Parameter estimates with 95% confidence intervals (CI) are provided.

|  | Parameter estimate [95% CI] | *t* value |
| --- | --- | --- |
| (Intercept) | 0.610 [0.282; 0.936] | 3.760 |
| A2 | 0.690 [0.326; 1.056] | 3.941 |
| DT2 | 0.296 [0.012; 0.580] | 2.059 |

Figure S1. *The effects of time of day and activity on proportion of observation time spent sleeping*

*Table S2*

Variables included in the final GLMM of proportion of observation time spent awake after first drowsiness. Parameter estimates with 95% confidence intervals (CI) are provided.

|  | Parameter estimate [95% CI] | *t* value |
| --- | --- | --- |
| (Intercept) | -1.459 [-1.928; -0.990] | -6.141 |
| TD1 | 0.482 [-0.270; 1.234] | 1.264 |
| TD2 | 1.686 [0.460; 2.912] | 2.714 |
| P2 | 0.397 [-0.045; 0.839] | 1.774 |
| P3 | 0.678 [0.236; 1.119] | 3.027 |
| P4 | 0.375 [-0.183; 0.934] | 1.326 |
| P5 | 0.766 [0.198; 1.335] | 2.660 |
| P6 | 0.576 [-0.006; 1.157] | 1.954 |
| DT2 | -0.440 [-0.777; -0.103] | -2.574 |
| A2 | -0.237 [-0.637; 0.163] | -1.170 |
| Loc2 | 0.283 [-0.136; 0.702] | 1.332 |
| A2:Loc2 | -0.578 [-1.139; -0.018] | -2.037 |

Figure S2a. *Location-specific effects of activity on proportion of observation time spent awake after first drowsiness*

**

Figure S2b. *The effects of time of day and period on proportion of observation time spent awake after first drowsiness*

Figure S2c. *The effects of technical difficulties on proportion of observation time spent awake after first drowsiness*

*Table S3*

Variables included in the final GLMM of proportion of observation time spent awake after first non-drowsiness sleep. Parameter estimates with 95% confidence intervals (CI) are provided.

|  | Parameter estimate [95% CI] | *t* value |
| --- | --- | --- |
| (Intercept) | -1.904 [-2.309; -1.499] | -9.794 |
| A2 | 0.278 [-0.235; 0.790] | 1.129 |
| Loc2 | 0.371 [-0.165; 0.907] | 1.443 |
| A2:Loc2 | -0.860 [-1.578; -0.143] | -2.496 |

Figure S3. *Location-specific effects of activity on proportion of observation time spent awake after first non-drowsiness*

*Table S4*

Variables included in the final MECM of latency to first drowsiness. Hazard ratios with 95% confidence intervals (CI) are provided.

|  | Hazard ratio [95% CI] | *z* value | *p* |
| --- | --- | --- | --- |
| A2 | 4.877 [1.225; 19.413] | 2.250 | 0.025 |

Figure S4. *The effect of activity on probability of first drowsiness after a given time elapsed*

*Table S5*

Variables included in the final MECM of latency to first non-drowsiness sleep. Hazard ratios with 95% confidence intervals (CI) are provided.

|  | Hazard ratio [95% CI] | *z* value | *p* |
| --- | --- | --- | --- |
| A2 | 3.047 [1.315; 7.062] | 2.600 | 0.009 |

Figure S5. *The effect of activity on probability of first non-drowsiness sleep*

*Table S6*

Variables included in the final GLMM of proportion of sleeping time spent in drowsiness. Parameter estimates with 95% confidence intervals (CI) are provided.

|  | Parameter estimate [95% CI] | *t* value |
| --- | --- | --- |
| (Intercept) | 0.300 [0.225; 0.376] | 7.928 |
| A2 | -0.085 [-0.145; -0.026] | -2.973 |
| DT2 | -0.098 [-0.143; -0.053] | -4.326 |
| Loc2 | -0.008 [-0.096; 0.081] | -0.175 |
| P2 | -0.005 [-0.083; 0.072] | -0.133 |
| P3 | 0.149 [0.072; 0.227] | 3.804 |
| P4 | 0.048 [-0.050; 0.145] | 0.972 |
| P5 | 0.040 [-0.058; 0.137] | 0.803 |
| P6 | 0.074 [-0.024; 0.171] | 1.492 |
| Loc2:P2 | 0.021 [-0.089; 0.130] | 0.375 |
| Loc2:P3 | -0.076 [-0.186; 0.033] | -1.372 |
| Loc2:P4 | -0.010 [-0.144; 0.124] | -0.144 |
| Loc2:P5 | 0.240 [0.106; 0.374] | 3.537 |
| Loc2:P6 | 0.116 [-0.026; 0.258] | 1.611 |

Figure S6. *The effects of activity and time of day on proportion of observation time spent in drowsiness*

*Table S7*

Variables included in the final GLMM of proportion of sleeping time spent in NREM. Parameter estimates with 95% confidence intervals (CI) are provided.

|  | Parameter estimate [95% CI] | *t* value |
| --- | --- | --- |
| (Intercept) | 0.287 [0.219; 0.355] | 8.392 |
| TD1 | -0.069 [-0.183; 0.044] | -1.208 |
| TD2 | -0.262 [-0.445; -0.077] | -2.816 |
| A2 | 0.123 [0.061; 0.184] | 4.156 |
| P2 | -0.067 [-0.132; -0.002] | -2.043 |
| P3 | -0.161 [-0.226; -0.096] | -4.905 |
| P4 | -0.110 [-0.192; -0.028] | -2.651 |
| P5 | -0.190 [-0.272; -0.108] | -4.573 |
| P6 | -0.214 [-0.299; -0.129] | -4.954 |
| DT2 | 0.111 [0.062; 0.161] | 4.455 |

Figure S7a. *The* *effect of activity on proportion of observation time spent in NREM*

Figure S7b. *The effects of technical difficulties on proportion of observation time spent in NREM*

*Table S8*

Variables included in the final ORM of observation time spent sleeping in REM. Odds ratios with 95% confidence intervals (CI) are provided.

|  | Odds ratio [95% CI] | *z* value | *p* |
| --- | --- | --- | --- |
| DT2 | 2.158 [1.123; 4.146] | 2.307 | 0.021 |
| A2 | 2.989 [1.169; 7.639] | 2.287 | 0.022 |

*Table S9*

Variables included in the final MECM of latency to first REM after the first drowsiness sleep. Hazard ratios with 95% confidence intervals (CI) are provided.

|  | Hazard ratio [95% CI] | *z* value | *p* |
| --- | --- | --- | --- |
| A2 | 1.692 [0.338; 8.462] | 0.640 | 0.520 |
| DT2 | 0.235 [0.056; 0.975] | -1.990 | 0.046 |
| A2:DT2 | 9.786 [1.538; 62.250] | 2.420 | 0.016 |

Figure S9. *Time of day-specific effects of activity on latency to REM after first drowsiness*

*Table S10*

Variables included in the final MECM of latency to first REM after the first non-drowsiness sleep. Hazard ratios with 95% confidence intervals (CI) are provided.

|  | Hazard ratio [95% CI] | *z* value | *p* |
| --- | --- | --- | --- |
| Loc2 | 0.140 [0.038; 0.512] | -2.970 | 0.003 |
| A2 | 1.341 [0.328; 5.480] | 0.410 | 0.680 |
| DT2 | 0.266 [0.072; 0.980] | -1.990 | 0.047 |
| A2:DT2 | 5.658 [1.033; 30.992] | 2.000 | 0.046 |

Figure S10a. *The effect of location on probability of REM after first non-drowsiness sleep*

Figure S10b. *Time of day-specific effects of activity on probability of REM after first non-drowsiness sleep*

**Exploratory Analyses on Sleep EEG Spectrum**

Data on sleep EEG power spectrum were derived and signal power spectrum was analyzed as a dependent variable of interest in models similar to those involving macrostructural variables. All methodological and procedural details were identical to those involving macrostructural variables, unless noted below.

**Method**

**Data analysis**

For sleep EEG power spectrum variables, artifact rejection was carried out manually on 4-s epochs before further automatic analyses. Average power spectral densities (1-30 Hz, 0.25 Hz bins) were calculated by a Fast Fourier Transformation (FFT) algorithm, applied to the 50% overlapping, Hanning-tapered 4 sec windows of the EEG signal of the Fz-Cz derivation, separately for each sleep phase (i.e., wake, REM, drowsiness, NREM). Relative power spectra were calculated separately for Drowsiness, NREM and REM sleep as proportion of total (1-30 Hz) power.

**Analytic Plan**

The same variables as in macrostructural analyses were examined as independent variables in sleep EEG power spectrum analyses, with the addition of: bandwidth as a single, 4-level factor with levels corresponding to the averaged power within each of four frequency ranges of delta (1-4 Hz), theta (4-8 Hz), alpha (8-12 Hz) and beta (12-30 Hz) and sleep phase (factor with three levels: REM, drowsiness and NREM). The dependent variable of interest was signal power spectrum.

In all above models estimating the effects of predictors on indices of sleep EEG power spectrum, the same variables were fixed factors with the same number of levels, with the exception of time of day (time of day had three levels, day- coded as 1, night1-coded as 2, and night2- coded as 3 without division into 1-hour periods) and period, and the addition of all three-, four-, and five-way interactions.

**Results**

Signal power spectrum was influenced by technical difficulties (χ^2^(2)=9.599, *p=*0.008 and interaction effects among activity, location, time of day, bandwidth, and phase, via: (1) a three-way interaction among time of day, bandwidth, and phase (GLMM; χ^2^(12)=37.506, *p<*0.001); (2) a four-way interaction among activity, location, time of day, and phase (χ^2^(4)=62.713, *p<*0.001); (3) a four-way interaction among activity, location, time of day, and bandwidth (χ^2^(6)=25.793, *p<*0.001); (4) and a four-way interaction among activity, location, bandwidth, and phase (χ^2^(6)=28.146, *p<*0.001, see online supplement, Table S11; Figure S11).

*Explanatory variables and factor levels in models of EEG power spectrum*

Time of day (DT): factor with three levels (day – 1; first 3h of night – 2; second 3h of night – 3)

Phase (PH): factor with three levels (REM – 1; drowsiness – 2; non-drowsiness – 3)

Bandwidth (BW): factor with four levels (alpha – 1; beta – 2; delta – 3; theta – 4)

Pre-sleep activity (A): factor with two levels (typical – 1; active – 2)

Locations (Loc): factor with two levels (home – 1; not home – 2)

Activity (A): factor with two levels (typical – 1; active – 2)

Technical difficulties (TD): factor with three levels (no – 0; minor – 1; major – 2)

*Table S11*

Variables included in the final General Linear Mixed Model of relative EEG power spectrum. Parameter estimates are provided.

|  | Parameter estimate | *t* value |
| --- | --- | --- |
| (Intercept) | -5.540 | -45.74 |
| TD1 | 0.063 | 42804 |
| TD2 | 0.019 | 0.65 |
| A2 | -0.048 | -0.32 |
| Loc2 | 0.217 | 1.38 |
| DTN1 | -0.161 | -1.96 |
| DTN2 | 0.101 | 42757 |
| PHS1 | -0.711 | -8.64 |
| PHS2 | -0.974 | -11.83 |
| BWbeta | -1.253 | -17.81 |
| BWdelta | 2.370 | 24.35 |
| BWtheta | 0.957 | 10.63 |
| A2:Loc2 | -0.271 | -1.29 |
| A2:DTN1 | 0.169 | 1.82 |
| A2:DTN2 | 0.217 | 2.31 |
| A2:PHS1 | -0.004 | -0.05 |
| A2:PHS2 | -0.103 | -1.11 |
| A2:BWbeta | 0.108 | 42765 |
| A2:BWdelta | -0.052 | -0.46 |
| A2:BWtheta | 0.106 | 1.00 |
| Loc2:DTN1 | 0.082 | 0.84 |
| Loc2:DTN2 | 0.071 | 0.73 |
| Loc2:PHS1 | -0.153 | -1.58 |
| Loc2:PHS2 | -0.227 | -2.34 |
| Loc2:BWbeta | -0.038 | -0.44 |
| Loc2:BWdelta | -0.415 | -3.47 |
| Loc2:BWtheta | -0.213 | -1.93 |
| DTN1:PHS1 | 0.357 | 42836 |
| DTN2:PHS1 | 0.317 | 3.55 |
| DTN1:PHS2 | 0.342 | 3.94 |
| DTN2:PHS2 | 0.192 | 42781 |
| DTN1:BWbeta | 0.051 | 0.62 |
| DTN2:BWbeta | 0.035 | 0.41 |
| DTN1:BWdelta | 0.095 | 0.82 |
| DTN2:BWdelta | -0.094 | -0.80 |
| DTN1:BWtheta | 0.041 | 0.39 |
| DTN2:BWtheta | -0.033 | -0.31 |
| PHS1:BWbeta | 0.113 | 1.34 |
| PHS2:BWbeta | -0.318 | -3.77 |
| PHS1:BWdelta | 0.627 | 5.37 |
| PHS2:BWdelta | 1.045 | 8.94 |
| PHS1:BWtheta | 0.172 | 1.60 |
| PHS2:BWtheta | 0.280 | 2.60 |
| A2:Loc2:DTN1 | 0.060 | 0.46 |
| A2:Loc2:DTN2 | -0.260 | -1.89 |
| A2:Loc2:PHS1 | 0.461 | 3.51 |
| A2:Loc2:PHS2 | 0.541 | 42837 |
| A2:Loc2:BWbeta | 0.101 | 0.87 |
| A2:Loc2:BWdelta | 0.357 | 42787 |
| A2:Loc2:BWtheta | 0.137 | 0.92 |
| A2:DTN1:PHS1 | -0.234 | -3.16 |
| A2:DTN2:PHS1 | -0.101 | -1.35 |
| A2:DTN1:PHS2 | -0.216 | -2.91 |
| A2:DTN2:PHS2 | -0.008 | -0.11 |
| A2:DTN1:BWbeta | -0.046 | -0.51 |
| A2:DTN2:BWbeta | 0.002 | 0.03 |
| A2:DTN1:BWdelta | -0.073 | -0.58 |
| A2:DTN2:BWdelta | -0.220 | -1.75 |
| A2:DTN1:BWtheta | -0.143 | -1.24 |
| A2:DTN2:BWtheta | -0.141 | -1.21 |
| A2:PHS1:BWbeta | -0.321 | -3.51 |
| A2:PHS2:BWbeta | -0.283 | -3.10 |
| A2:PHS1:BWdelta | 0.148 | 42753 |
| A2:PHS2:BWdelta | 0.177 | 1.41 |
| A2:PHS1:BWtheta | -0.040 | -0.34 |
| A2:PHS2:BWtheta | -0.010 | -0.09 |
| Loc2:DTN1:PHS1 | -0.064 | -0.83 |
| Loc2:DTN2:PHS1 | -0.127 | -1.64 |
| Loc2:DTN1:PHS2 | -0.145 | -1.87 |
| Loc2:DTN2:PHS2 | 0.162 | 42774 |
| Loc2:DTN1:BWbeta | 0.078 | 0.83 |
| Loc2:DTN2:BWbeta | 0.077 | 0.82 |
| Loc2:DTN1:BWdelta | -0.027 | -0.21 |
| Loc2:DTN2:BWdelta | -0.043 | -0.33 |
| Loc2:DTN1:BWtheta | -0.048 | -0.40 |
| Loc2:DTN2:BWtheta | -0.065 | -0.54 |
| Loc2:PHS1:BWbeta | -0.071 | -0.76 |
| Loc2:PHS2:BWbeta | 0.010 | 0.11 |
| Loc2:PHS1:BWdelta | 0.310 | 2.37 |
| Loc2:PHS2:BWdelta | 0.300 | 2.29 |
| Loc2:PHS1:BWtheta | 0.128 | 42740 |
| Loc2:PHS2:BWtheta | 0.188 | 1.55 |
| DTN1:PHS1:BWbeta | -0.108 | -1.43 |
| DTN2:PHS1:BWbeta | -0.008 | -0.11 |
| DTN1:PHS2:BWbeta | -0.010 | -0.13 |
| DTN2:PHS2:BWbeta | 0.177 | 42787 |
| DTN1:PHS1:BWdelta | -0.127 | -1.21 |
| DTN2:PHS1:BWdelta | -0.213 | -1.93 |
| DTN1:PHS2:BWdelta | -0.138 | -1.32 |
| DTN2:PHS2:BWdelta | -0.202 | -1.83 |
| DTN1:PHS1:BWtheta | 0.006 | 0.07 |
| DTN2:PHS1:BWtheta | -0.031 | -0.31 |
| DTN1:PHS2:BWtheta | -0.018 | -0.19 |
| DTN2:PHS2:BWtheta | -0.095 | -0.93 |
| A2:Loc2:DTN1:PHS1 | -0.267 | -2.58 |
| A2:Loc2:DTN2:PHS1 | 0.345 | 42807 |
| A2:Loc2:DTN1:PHS2 | -0.211 | -2.04 |
| A2:Loc2:DTN2:PHS2 | -0.331 | -3.00 |
| A2:Loc2:DTN1:BWbeta | -0.311 | -2.47 |
| A2:Loc2:DTN2:BWbeta | -0.201 | -1.50 |
| A2:Loc2:DTN1:BWdelta | 0.151 | 0.87 |
| A2:Loc2:DTN2:BWdelta | 0.165 | 0.89 |
| A2:Loc2:DTN1:BWtheta | 0.166 | 42738 |
| A2:Loc2:DTN2:BWtheta | 0.179 | 42740 |
| A2:Loc2:PHS1:BWbeta | 0.188 | 1.45 |
| A2:Loc2:PHS2:BWbeta | -0.019 | -0.15 |
| A2:Loc2:PHS1:BWdelta | -0.507 | -2.82 |
| A2:Loc2:PHS2:BWdelta | -0.385 | -2.15 |
| A2:Loc2:PHS1:BWtheta | -0.167 | -1.00 |
| A2:Loc2:PHS2:BWtheta | -0.243 | -1.46 |

Figure S11. *Interaction effect of activity, location, time of day, and bandwidth on relative EEG power spectrum across the different sleep stages:* Activity, location, time of day, bandwidth, and phase had interaction effects on signal power spectrum.
